# Towards lasing systems for distributed fibre sensing

**DOI:** 10.1038/s41377-026-02217-5

**Published:** 2026-02-24

**Authors:** Luc Thévenaz

**Affiliations:** EPFL - Ecole Polytechnique Fédérale de Lausanne, Institute of Electrical and Micro Engineering, Station 11, 1015 Lausanne, Switzerland

**Keywords:** Imaging and sensing, Optical metrology

## Abstract

A novel concept for distributed fiber sensing has recently been introduced, in which the sensing fiber itself forms a laser cavity. This configuration yields a high-quality response that is largely independent of position, thereby enhancing measurement accuracy and acquisition speed, and opening new avenues towards higher-performance sensing technologies.

## Abstract (web summary)

Synchronous pumping enables laser-based distributed fiber sensors to achieve uniform signal quality regardless of distance, addressing traditional range limitations and advancing flexible, high-performance sensing capabilities.

## Graphical abstract

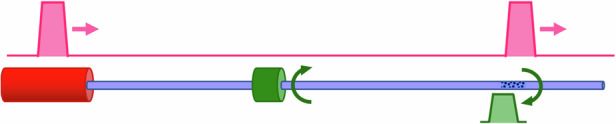

Distributed optical fiber sensing has become a key technology for monitoring optical transmission lines and assessing the integrity and characteristics of critical infrastructure^1^. Such sensors play a central role in enabling more efficient and reliable system operation, supporting the societal demand for safer environments and a more sustainable use of resources.

These sensors exploit the weak, continuous reflections arising from scattering processes in silica fibers. In most cases, position-resolved information is obtained using time-domain techniques: an intense light pulse is launched into the fiber, and the tiny continuous backscattering is recorded. The temporal profile of the signal is then mapped to position through the time of flight. Alternatively, frequency-domain methods can be used, taking advantage of coherent detection schemes that are particularly effective for short-range interrogation with very high spatial resolution. A third approach—the correlation-domain technique^2,3^—achieves spatial localization through a clever modulation scheme: the continuous reflection from a modulated input light is multiplied before detection by a delayed replica carrying the same modulation. By varying the delay, different positions along the fiber can be addressed when correlation is maximized.

Although these techniques have all demonstrated high relevance and are approaching their ultimate performance, fiber loss remains the major limiting factor^4^. It constrains the sensing range and causes the signal quality to degrade with distance, leading to the lowest signal-to-noise ratio at the far end of the fiber.

To overcome this position-dependent degradation, researchers have explored ways to make the sensor itself a lasing device—using the sensing fiber as part of the laser cavity. In a laser cavity, the light intensity is naturally fairly uniform along its length, mitigating signal variation with distance. Moreover, if the sensing information is encoded in the spectral response, the laser will emit at the wavelength of maximum reflectivity, making the sensing information directly accessible through the emission wavelength.

The idea has been circulating since the 1990s, though without notable progress until 2010, when a pioneering study demonstrated a Raman-based system in which the sensing fiber formed the cavity of a Raman laser^5^. This configuration provided a distributed, quasi-uniform amplification of the sensing signal, precisely compensating for fiber loss. While the sensing signal itself did not strictly lase, the system delivered a remarkably uniform response over a 50-km fiber.

Achieving true lasing of the sensing signal, however, introduces substantial complexity. The distributed reflectivity arising from scattering is extremely weak, and the interaction must also be localized along the fiber. A breakthrough came in 2022, when Murray et al. proposed a Brillouin-based scheme employing synchronous pumping^6^: pump pulses were launched at a repetition rate precisely matching the round-trip propagation time of a loop cavity formed by the sensing fiber, as shown in Fig. 1a. Under this condition, light scattered from a given position was coherently reinforced at each round trip until laser oscillation occurred. The resulting time-domain distribution of lasing frequencies directly mapped the spatial variation of the Brillouin frequency shift along the fiber. Because laser gain saturation inevitably depletes the pump, the accessible sensing range can shrink considerably, and the system appears limited to interactions involving stimulated backward scattering.

**Fig. 1:**
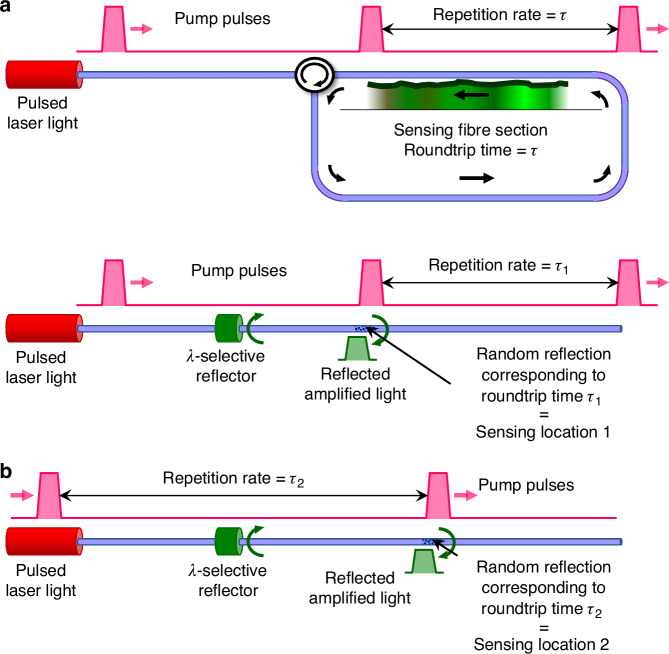
Proposed configurations for lasing-based distributed fiber sensors. **a** Configuration introduced in ref. ^6^, where the sensing fiber forms a closed loop and pump pulses are launched at a repetition rate matched to the loop round-trip time. This architecture is inherently limited to stimulated backward scattering. Its main advantage is that it provides a fully distributed measurement in a single temporal trace, but it is prone to pump depletion inherent to laser-gain saturation, which can make the usable sensing range substantially reduced. **b** Configuration introduced in ref. ^7^, in which the sensing section is linear and operates through distributed reflective scattering, making it compatible with any process featuring a spectral reflectivity peak. This scheme requires a wavelength-selective reflector with high reflectivity at the lasing wavelength and high transmission at the pump wavelength. Only one sensing position can be probed per acquisition—defined by the pulse-repetition rate, as illustrated by the two examples shown—which inherently suppresses the impact of pump depletion on the sensing range, but substantially increases the total time required to acquire a full trace

In a recent paper in *Light: Science & Applications*^7^, Tovar et al. propose a different method for localizing reflection, in which any position can be addressed independently. Their configuration also requires access to only one end of the sensing fiber, in contrast to the loop-cavity approach.

The system again relies on synchronous pumping, but now the cavity is linear, with a strong reflector at the near end and the weak distributed backscattering of the fiber acting as the far-end reflector, as shown in Fig. 1b. By tuning the repetition rate of the pump pulses, only reflections from the position satisfying the round-trip resonance condition are amplified on successive passes. The concept is both novel and flexible: nearby positions can be interrogated at high repetition rates for fast acquisition, whereas distant points require slower repetition rates. One intrinsic limitation remains—the usable range of repetition rates must not exceed one octave to avoid ambiguity from amplification at integer fractions of the repetition rate.

Tovar et al. demonstrated their concept using coherent Rayleigh scattering assisted by Kerr parametric amplification through modulation-instability sidebands. Although the experiment covered only a 1-km sensing range and required a fiber with enhanced distributed reflectivity, the proof-of-concept is compelling. Remarkably, information can be collected from either end of the fiber.

There remains ample room for improvement, and a combination of these two recent approaches could lead to highly performant systems. For example, the flexible configuration proposed by Tovar et al. could likely be adapted for Brillouin-based distributed sensing. It should be emphasized that the advantage of these lasing configurations does not primarily lie in acquisition speed—many cavity round-trips are needed to build up the lasing signal, often comparable to the number of averages required in conventional schemes—but rather in the fact that the lasing output directly carries the relevant spectral information, eliminating the need for further processing. This innovative concept illustrates that new avenues in distributed sensing continue to emerge—and that there is still plenty of room for creativity and progress in this field.
